# Dietary Phytochemicals Targeting Cancer Stem Cells

**DOI:** 10.3390/molecules24050899

**Published:** 2019-03-04

**Authors:** Alena Liskova, Peter Kubatka, Marek Samec, Pavol Zubor, Milos Mlyncek, Tibor Bielik, Samson Mathews Samuel, Anthony Zulli, Taeg Kyu Kwon, Dietrich Büsselberg

**Affiliations:** 1 Clinic of Obstetrics and Gynecology, Jessenius Faculty of Medicine, Comenius University in Bratislava, Martin, 03601 Bratislava, Slovakia; alenka.liskova@gmail.com (A.L.); marek.samec@gmail.com (M.S.); Pavol.Zubor@jfmed.uniba.sk (P.Z.); tbielik57@gmail.com (T.B.); 2 Department of Medical Biology, Jessenius Faculty of Medicine, Comenius University in Bratislava, Martin, 03601 Bratislava, Slovakia; 3 Department of Obstetrics and Gynecology Faculty Hospital Nitra Constantine the Philosopher University, 949 01 Nitra, Slovakia; mlyncekmilos@hotmail.com; 4 Department of Physiology and Biophysics, Weill Cornell Medicine-Qatar, Education City, Qatar Foundation, P.O. Box 24144, Doha 24144, Qatar; sms2016@qatar-med.cornell.edu; 5 Institute for Health and Sport, Victoria University, Melbourne, VIC 3011, Australia; Anthony.Zulli@vu.edu.au; 6 Department of Immunology and School of Medicine, Keimyung University, Dalseo-Gu, 426 01 Daegu, Korea; kwontk@dsmc.or.kr

**Keywords:** cancer stem cells, phytochemicals, plant-derived foods, fruit, vegetable, cell signaling

## Abstract

There is an increasing awareness of the importance of a diet rich in fruits and vegetables for human health. Cancer stem cells (CSCs) are characterized as a subpopulation of cancer cells with aberrant regulation of self-renewal, proliferation or apoptosis leading to cancer progression, invasiveness, metastasis formation, and therapy resistance. Anticancer effects of phytochemicals are also directed to target CSCs. Here we provide a comprehensive review of dietary phytochemicals targeting CSCs. Moreover, we evaluate and summarize studies dealing with effects of dietary phytochemicals on CSCs of various malignancies in preclinical and clinical research. Dietary phytochemicals have a significant impact on CSCs which may be applied in cancer prevention and treatment. However, anticancer effects of plant derived compounds have not yet been fully investigated in clinical research.

## 1. Introduction

Despite progress in anticancer therapy, cancer is a major health problem and one of the leading causes of morbidity worldwide [1,2]. Cancer is characterized by uncontrolled cell growth, invasiveness, and formation of metastasis. Malignant tumours are represented by heterogeneous populations of cancer cells. The heterogeneity may be explained by evolutional accumulation of mutations in one or few cells or by presence of cells with stem-like properties. Cancer stem cells (CSCs) are characterized as a subpopulation of cells with an intrinsic ability of self-renewal and differentiation [3,4,5,6]. Dietary phytochemicals are suggested to possess anti-cancer properties with minimal or no side effects [7,8,9]; moreover, they may also improve the efficacy of chemo- or radiotherapy, and therefore may represent an important strategy to target CSCs [10].

### 1.1. Aim of the Study

The review focuses on the anticancer effectiveness of dietary phytochemicals, either isolated or as mixtures via targeting CSCs. Firstly, it discusses the basics of CSCs and signaling pathways modulating their stem-like properties. The core of the review is the summary of preclinical and clinical studies evaluating whether dietary phytochemicals target CSCs in various malignancies. Plant-derived dietary compounds which are effective agents against CSCs in preclinical in vitro and in vivo research should be further evaluated in clinical research. We emphasize the need to include dietary phytochemicals in the current clinical research.

### 1.2. Source of Data

Data were recovered from the biomedical literature published in the English-language literature by use of “cancer stem cells” and “plant-based functional foods“ or “phytochemicals” or “fruit“ or “vegetables” or “herbs” as either a keyword or medical subject heading (MeSH) term in searches of the PubMed bibliographic database. We emphasize the most recent scientific papers from the years 2013–2019. About 40 studies were selected with the database accessed between December 2018 and February 2019.

## 2. CSCs (Cancer Stem Cells)

CSCs are multipotent cells exhibiting stem-like properties and possessing the capability of the initiation of tumor growth, invasiveness, and dissemination to distant organs [6,11]. CSCs are like normal stem cells in several ways; however, if the balance of the influence of internal or external factors is not maintained, it may lead to hyperproliferation and metastasis [12]. Moreover, CSCs are resistant to chemical and electromagnetic insults due to their infrequent replication, active drug efflux system, increase in defense against reactive oxygen species and importantly higher ability of DNA repairs which may result in a lower rate of apoptosis [13]. Moreover, autophagy is characterized as a process allowing cells to survive under stress conditions. Accordingly, therapy-resistance and survival of CSCs may be also associated with increase in autophagy activity [14]. CSCs are also characterized by active telomerase expression, increased membrane transport activity [2] and hypoxic niche [12]. The survival of stem cells and CSCs depends on their niche or microenvironment which provides signals regulating their proliferative and self-renewal maintenance [15]. In conclusion, CSCs are related to the development of cancer, metastasis and resistance to conventional anticancer therapies and recurrence or relapse of malignancy. Targeting CSCs is a promising strategy of anti-cancer research [12,16].

### 2.1. Cancer Stem Cells Markers

**Figure 1:**
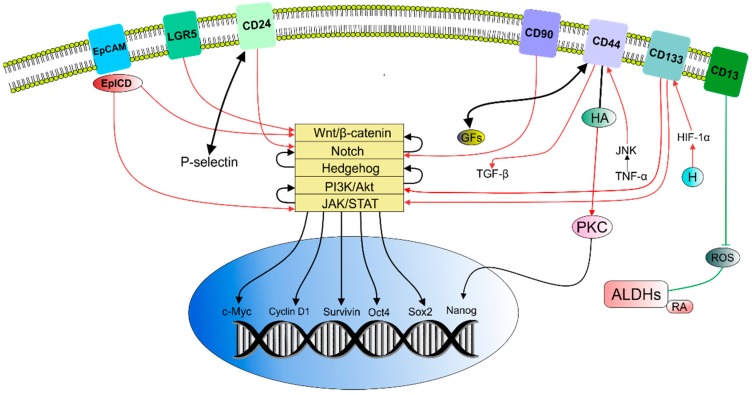
Association between surface markers and promotion of CSCs stem-like properties [3,20,23,24,25,26,27,28,29,30,31,32,33,34,35,36,37,38,39,40,41].

CSCs have been recognized in various types of tumors and it is now possible to identify and isolate them using a distinctive profile of surface (e.g. CD24, CD44, CD133, CD49, CXCR4, LGR5) or intracellular markers (e.g. ALDH) [17]. Table 1 lists a brief overview of generally accepted markers associated with CSCs. However, no universal marker for CSCs has been identified [13]. Moreover, their phenotypes exhibit different markers due to the occurrence of epigenetic alterations, possible presence of multiple CSCs pools or technical variations [18]. Importantly, these markers are also present in normal stem cells and other cell types [19]; therefore, a combination of markers is usually required to denote the CSCs population [20]. CSCs markers are also associated with induction of stem-like phenotype of cancer cells via modulation of various molecular pathways (Figure 1). On the contrary, a novel 3D spheroid-based label-retention assay followed by FACS sorting allows the identification and isolation of stem cells at a single cell level which is considered to be a marker-free method of isolation of both normal stem cells and CSCs. Consequently, this method dealing with isolation and characterization of the novel properties of CSCs may be of a great interest in anti-cancer therapeutic strategy [14]. Moreover, the isolation of stem cells can be also helpful in the understanding of the initiation of carcinogenesis as a result of environmental carcinogens targeting stem cells as was demonstrated by Hu et al. [21] and Prins et al. [22] in in vivo models using prostasphere cells.

Cluster of differentiation 44 (CD44) promotes increase in growth factor beta (TGF-β) leading to Epithelial–mesenchymal transition (EMT). Binding of hyaluronic acid (HA) with CD44 activates Protein kinase C (PKC) which then phosphorylates transcription factor Nanog resulting in upregulation of ATP binding cassette subfamily B member 1 (ABCB1) contributing to multidrug resistance (MDR)). CD44 serves as a coreceptor for growth factors and stimulate CSCs self-renewal. Tumour necrosis factor alfa (TNF-α) upregulates CD44 through Janus kinase (JNK), thus inducing migration, invasion, metastasis or EMT. Cluster of differentiation 24 (CD24) stimulates metastasis formation via interaction with P-selectin and cancer progression and trigger EMT via activation of Notch1signaling. Cluster of differentiation 133 (CD133) is involved in tumour cell proliferation, metastasis, tumorigenesis or therapy resistance—via activation of the phosphatidylinositol-3-kinase (PI3K)/Akt. Hypoxia (H) in stem cells and the tumour microenvironment promote CD133 expansion via upregulation of hypoxia-inducible factor 1-alpha (HIF-1α). Overexpression of CD133 is associated with tumour progression through epidermal growth factor receptor (EGFR)-dependent Akt activation. The role of cluster of differentiation 90 (CD90) in cancer depends on the cancer type and signaling mechanism. For example, cancer stem-like activity is elevated through up-activation of Notch pathway. Increased expression of cluster of differentiation13 (CD13) reduces reactive oxygen species (ROS) promoting CSCs survival via EMT. Leucine-rich repeat-containing G-protein-coupled 5 (LGR5) promotes proliferation of cancer cells via activation of Wnt/β-catenin pathway. Epithelial cell adhesion molecule (EpCAM) cleavages with its intracellular domain (EpICD) and provide key signals for achieving CSCs properties by modulation of Wnt pathway or LIF/STAT3. Activity of aldehyde dehydrogenase (ALDH) may protect CSCs against cell death caused by ROS. ALDHs metabolizes retinoic acid (RA) thus regulating stem-like properties of CSCs. Aberrantly regulated signaling pathways and cross-talks between them may ultimately influence their target genes such as c-Myc, cyclinD1, Survivin, Nanog, Oct-4, Sox2, etc.

### 2.2. EMT (Epithelial–Mesenchymal Transition)

EMT is the reversible change occurring usually during embryogenesis in which epithelial cells acquire mesenchymal phenotypes [42]; moreover, EMT is an important process during wound healing and reestablishment of basal and apical polarity [20]. However, EMT is a crucial step in the initiation of tumor metastasis in which cancer cells alter their morphology, loose epithelial cell-cell junctions and obtain migratory properties and metastatic capacity. CSCs are considered to possess enhanced EMT ability, and thus produce metastatic tumors or circulate in the body in a dormant state, until its activation which may occurs years later [2,20]. EMT also supports the resistance of CSCs to therapeutic drugs [6]. The process of EMT is associated with modulation of E-cadherin, N-cadherin, vimentin, C-X-C chemokine receptor type 4 (CXCR4) or cyclooxygenase-2 (COX-2) and also with activation of transcription factors Snail or Twist [20,43]. It is indicated that metastatic cancer cells undergoing EMT may possess a CSCs phenotype [44].

### 2.3. Molecular or Cellular Signaling of CSCs

Strictly regulated signaling pathways control the activity of stem cells [6]. However, some pathways could be abnormally repressed or activated in human malignancies and these irregularities contribute to the proliferative, differentiation, self-renewal, and survival properties of CSCs via aberrant regulation of target genes, such as c-Myc, cyclin D1, Survivin, Nanog, Oct-4, Sox2 and others. Interestingly, signaling pathways are not linear which means that cross-talk between various pathways occasionally occur and may lead to consequences including promotion of resistance to therapy, CSCs expansion or other fatal consequences [40,41].

#### 2.3.1. Signaling Pathways in CSCs

The evolutionary conserved Notch signaling pathway possesses an important role in the balance of differentiation, cell cycle progression [6], survival, and apoptosis of stem cells. Alterations in this ligand-receptor pathway guide undifferentiated cells toward malignant transformation and acquisition of EMT via increased expression of Notch1, Sox2, Nanog, Oct4 or Lin28; moreover the cross-talks between Notch and other oncogenic signaling pathways also play a crucial role in the development of cancer [41,45]. Notch signaling is considered to be one of the most activated pathways in cancer cells and is an important linkage between angiogenesis and CSCs self-renewal [46].

Wnt signaling pathway is involved in embryonic development and homeostasis of tissues. Evolutionary conserved and highly complex Wnt signaling is considered to encompass two pathways which are not exclusive and cross-talk may occur between them. β-catenin-independent pathway (or noncanonical) with calcium as the major mediator regulates asymmetrical division of cells, cell polarity and migration [40,45]. On the contrary, the transcriptional regulator β-catenin-dependent (canonical) pathway regulates the survival and proliferation of cells [47]. The aberrant activation of Wnt pathway and deregulated expression of Wnt-binding proteins, ligands, inhibitors or other co-regulators is associated with various human malignancies, acquisition of EMT phenotype, self-renewal or cancer cell dedifferentiation into CSCs. Targeting Wnt may be another option of decreasing or eliminating CSCs [41,45].

The Hedgehog (HH) signaling pathway plays a crucial role in the embryonic development, especially the development of skin, hair follicles and sebaceous glands and also in adult brain development [6]. Moreover, HH functions in the regulation of proliferation, maintenance of the stem cells and progenitor cell and self-renewal capacity [48]. There are several Hedgehog homologues which are well studied in mammals including sonic (sHH), desert (dHH), and indian (iHH). Significantly, it is supposed that HH plays an important role in the process of acquiring stem cell-like properties during the EMT [41]. For instance, aberrant activation of sHH/transcription factor GLI1 signaling may lead to changes in expression of GLI1-regulated oncoproteins like N-myc, CyclinD1, Foxm1, and Bcl-2 playing a crucial role in CSCs self-renewal [49].

The phosphatidylinositol-3-kinase (PI3K)/Akt and the mammalian target of rapamycin (mTOR) is a signaling pathway playing crucial role in metabolism, proliferation, angiogenesis, differentiation, and survival of cells [48]. It is abnormally regulated in cancer cells due to mutation, deletion, methylation, amplification or post-translational modifications and is important for regulation of apoptosis, radioresistance, metastasis, and maintenance of CSCs populations. Phosphatase and tensin homolog (PTEN) is suggested to be a negative regulator of PI3K/Akt/mTOR pathway and also to function as a tumour suppressor [50]. Components of PI3K pathway are considered to be the most activated and mutated in human cancers, thus it is important to consider its targeting in cancer treatment [51].

The Janus kinase (JAK) and signal transducer and activator of transcription (STAT) signaling pathway possess and important role in cytokines and growth factor signaling affecting cell growth, proliferation and immune response. Aberrant regulation of the JAK/STAT pathway is associated and implied with maintenance of germ-line stem cell populations in various cancers [48]. Moreover, interleukin-8 (IL-8) is suggested to trigger the activation of STAT3 which may lead to inflammation, ROS production and multidrug resistance (MDR) [52]. More detailed overview of aberrantly regulated CSCs signaling pathways attributed to various malignancies is shown in Table 2. Importantly, this table is adjusted to several concrete cancer types, and thus other abnormally regulated pathways or signaling molecules, which are not mentioned, may be also responsible for the CSCs-phenotype of other malignancies.

Abberant regulation of Notch signaling may be modulated via abnormal expression of Notch ligands including Delta-like (DLL1/3/4) and JAGGED (JAG1/2), Notch receptors (Notch1–4) or Notch target genes (Hes1). Wnt/β-catenin pathway may contribute to cancer-like phenotype of cells via abnormal expression of Secreted frizzled-related proteins (SRFP-1), Wnt inhibitory factor (WIF), Dickkopf-related protein (DKK), Axis inhibition protein 2 (AXIN2) and increased levels of Wnt signaling proteins including Lymphoid enhancer-binding factor 1 (LEF-1) or T-cell factor 4 (TCF-4) binding to which β-catenin influence expression of target genes (eg. Cyclin D or c-Myc). Deregulation of Hedgehog (HH) pathway may be influenced via aberrant expression of HH ligands (sonic, desert, indian), receptors PATCHED (PTCH), transmembrane proteins SMOOTHENED (SMO) or transcription factors Zinc finger proteins (GLI1-3). Abnormal modulation of PI3K/Akt/mTOR may be based on dysregulated Protein kinase B (Akt) or negative regulator of the Phosphatase and tensin homolog (PTEN). JAK/STAT signaling may be deregulated via abnormal expression of Signal transducer and activator of transcription 3 (STAT3).

#### 2.3.2. Apoptotic and Death Resistance Signaling of CSCs

Apoptosis is regulated by an extrinsic or intrinsic pathway. Dysregulated apoptosis is a hallmark of cancer, and failure in signaling either of extrinsic or intrinsic pathways occurs also in CSCs [2,55]. Deficiency in apoptotic pathways of CSCs is induced via various mechanisms. Firstly, upregulation of cFLIPs, FLICE-like inhibitory proteins functioning as negative modulators of death receptor-induced apoptosis, and dysregulated expression of Bcl-2 family members are critical for the survival of CSCs. Additionally, increase in expression of inhibitors of apoptosis proteins (IAPs) is also associated with the tendency of CSCs to evade apoptosis. An increase in survivin, antiapoptotic protein which belongs to IAP family, may also contribute to maintenance of CSCs [55,56]. Furthermore, it is suggested that chemo- or radioresistance of CSCs is dependent on interleukin-4 (IL-4) signaling pathway because upregulation of the already mentioned pathway may result in apoptosis resistance [6]. Increase in autophagy flux is also observed in stem cells; therefore it may be related with therapy resistance of CSCs (Hu et al. [14]). Cancer cells may contain activated nuclear factor-κB (NF-κB) and tumour necrosis factor receptor-1 associated death domain protein (TRADD) which is considered to possess an important role in NF-κB activation and survival of CSCs [56]. NF-κB enables CSCs to mediate metastasis [40] and cooperates with other pathways which are associated with CSCs, such as Notch, TGF-β or STAT3 [57]. Importantly, activation of nuclear respiratory factor 2 (Nrf-2) may lead to inhibition of NF-κB signaling [58]. In addition, overactivation of ATP-binding (ABC) efflux multidrug resistance transporters, which induce resistance to chemotherapy, was observed to be highly expressed in various CSCs. Moreover, hypoxia-inducible factor 1 (HIF-1) mediating efflux of chemotherapy is implicated in resistance of CSCs to chemotherapy. The use of agents reducing efflux may be a viable strategy targeting CSCs. Activation of enzyme aldehyde dehydrogenase (ALDH) is associated with drug metabolic activities. Restoration of apoptotic processes in CSCs may lead to increased sensitiveness of CSCs to anticancer strategies [56,59].

**Figure 2:**
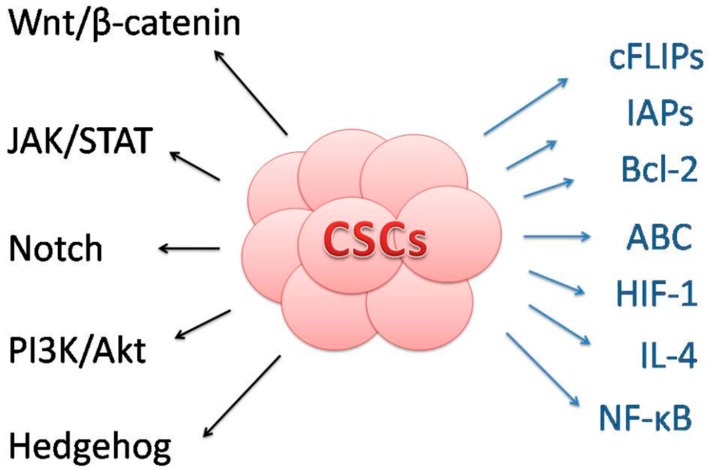
Mechanisms involved in stem-like maintenance and death resistance of CSCs.

In conclusion, multiple mechanisms are involved in regulation of the stem-cells related processes of self-renewal, differentiation or apoptosis, many of them are deregulated in CSCs. Figure 2 depicts an overview of signaling pathways involved in the maintenance of CSCs survival, self-renewal, differentiation, and mechanisms of death resistance. Nevertheless, several pharmaceuticals and dietary phytochemicals were suggested to repair these abnormally regulated cellular or molecular signaling pathways in CSCs, and thus function as anticancer agents. In the following chapter we will focus on anticancer effects of dietary phytochemicals (isolated and/or compounds) in CSCs.

Self-renewal, differentiation and maintenance of other stem-like properties may be mediated via modulation of signaling pathways including Notch, Wnt/β-catenin, Hedgehog, PI3K/Akt, JAK/STAT and others. cFLIPS, FLICE-like inhibitory proteins; IAPs, inhibitors of apoptosis proteins; Bcl-2, Bcl-2 family proteins; ABC, ATP-binding efflux multidrug resistance transporters; HIF-1, hypoxia-inducible factor 1; IL-4, interleukin-4; NF-κB, nuclear factor-κB are suggested to modulate CSCs resistance to death and cancer therapies.

## 3. Natural Compounds Targeting CSCs in Cancer Research

The importance of natural compounds in cancer treatment or prevention is supported by abundant evidence from cancer research [60]. Natural products are a source of bioactive compounds [20] demonstrating antioxidant, proapoptotic, and antiproliferative effects on a variety of cancers, for which existence of CSCs have been reported [61]. Significantly, dietary phytochemicals possess an ability of multilateral targeting of cellular and molecular signaling pathways which are abnormally activated in CSCs [18,59]. Since the first identification of CSCs in the late 1990s, there is great interest of this approach in cancer research [15]. Although, the clear evidence of phytochemicals targeting CSCs was in the early 21^st^ lacking, a possible association between phytochemicals and their potential anti-CSCs effects was indicated by several studies. Jaiswal et al. [62] and Ryu et al. [63] suggested an active role of curcumin in the Wnt/β-catenin attenuation in colon cancer cells. Moreover, downregulation of Notch1 was observed in pancreatic cancer cells [64]. However, more recent evidence from various preclinical and clinical studies suggests a direct association of natural bioactive compounds either in form of isolated dietary phytochemicals or in a form of plant functional foods in targeting CSCs.

### 3.1. Preclinical Research

#### 3.1.1. Isolated Phytochemicals

Epigallocatechin-3-gallate (EGCG) is the main constituents of green tea and its protective effects are associated with various human malignancies whereas the combination of EGCG and anticancer therapy is more effective for inhibiting CSCs [65]. Interestingly, EGCG diminished lung CSCs activity in vitro via inhibition of tumorsphere formation, decrease in CSCs markers, suppression of proliferation, and induction of apoptosis. The mechanism of already mentioned effects of EGCG is attributed to the modulation of the Wnt/β-catenin pathway [66]. EGCG also inhibited self-renewal capacity of head and neck CSCs by attenuating the expression of stem cell markers and suppressing sphere forming capacity. Moreover, EGCG augmented cisplatin-mediated chemosensitivity by suppression of ABC transporter genes, inhibited tumor formation and induced apoptosis in xenograft model. The presumed mechanism of the anti-CSCs activity of EGCG is attributed to decrease in transcription level of Notch signaling components [67].

Resveratrol is a protective ingredient widely spread in the traditional Mediterranean diet which is considered to lower the risk of cancer. Resveratrol and its analogue pterostilbene target CSCs via multiple signaling pathways [68]. Resveratrol is characterized as polyphenolic stilbene derivate found in the skin of grapes and berries which possess antioxidant, anti-inflammatory, and anticarcinogenic properties [20]. Resveratrol suppressed the Wnt/β-catenin signaling pathway in breast CSCs in vitro and in vivo, and thus inhibited breast cancer stem cells and induced autophagy [69]. Moreover, resveratrol was found to impair glioma stem cells proliferation and motility by modulating the Wnt signaling and EMT activators in glioblastoma multiforme lines [70]. Furthermore, resveratrol eliminated CSCs of osteosarcoma by reduction of expression of cytokines activating JAK/STAT signaling [71]. Pterostilbene is a bioactive compound of blueberries and grapes gaining an attention due to its chemo-preventive effects in a variety of cancer types. Tumor-associated macrophages (TAM) are suggested to promote metastasis and malignancy; interestingly, it is suggested that pterostilbene influences CSCs/TAM regulation in breast cancer. Pterostilbene suppressed the generation of breast CSCs via modulation of NF-κB/microRNA 448 circuit [72]. Moreover, pterostilbene was found to function as an anticancer stem cell agent via suppressing irradiation-mediated enrichment of CD133+ Mahlavu cells, preventing tumor sphere formation, reducing stemness gene expression and suppressing invasion, migration, and process of EMT in hepatoma CSCs [43].

Genistein is a soy isoflavone functioning as a natural NF-κB inhibitor [73]. Genistein is associated with antitumor effects in various malignancies, especially in breast and prostate cancer. The study evaluating effects of genistein in vitro and in vivo was performed in MCF-7 breast cancer cells and in nude mice. Genistein inhibited breast cancer stem cells through down regulation of Hedgehog-GLI1 signaling pathway [74]. Moreover, genistein was found to inhibit stemness of SKOV3 cells induced by macrophages co-cultured with ovarian cancer stem-like cells thus becoming a potential chemo-preventive agent in human ovarian cancer. Genistein disrupted interaction between OCSLCs and THP-1 macrophages via blocking IL-8/STAT3, reversing M2 polarization of macrophages and inhibiting stemness of SKOV3 cells in co-culture system and co-injection in nude mice [52]. 7-Difluoromethoxyl-5,4′-di-*n*-octyl genistein (DFOG) is a novel synthetic genistein analogue. DFOG inhibited stem-like properties and reverse EMT phenotype in gastric cancer stem-like cells in vitro [75].

Curcumin is a dietary polyphenol derived from *Curcuma longa*. Curcumin targets CSCs through acting on the signaling pathways including Wnt, HH or Notch [76,77]. Importantly, curcumin decreased CSCs markers in Burkitt lymphoma and acute myeloid leukemia cell line via modulation of self-renewal CSCs mechanisms [78]. Another study evaluated effects of curcumin on the reduction of breast CSCs population for sensitizing cancer cells to mitomycin C. Curcumin sensitized breast cancer cells to chemotherapy via decrease in ABC transporter (ABCG2) expression [79]. Curcumin also suppressed malignant glioma cells growth and induced apoptosis via inhibition of sHH/Gli1 pathway in vitro and in vivo [49]. Curcumin also induced cell cycle arrest via regulation of G0/G1 phase related factors including cyclin D1, cyclin-dependent kinase 2 (CDK-2), nuclear factor erythroid 2–related factor 2 (p21) and cyclin-dependent kinase inhibitor 1 (p27) and apoptosis in prostate cancer cells in vitro via down regulation of Notch signaling [80].

Isothiocyanates (ITCs) suppress cellular proliferation, EMT and self-renewal of CSCs via inhibition of oncogenic signaling pathways such as NF-κB, STAT3 or other pathways which are found to be upregulated in various cancers [81]. Phenethyl isothiocyanate (PEITC) and sulforaphane are most widely investigated isothiocynates of cruciferous plants [82]. Sulforaphane reduced tumor growth of orthotopically implanted primary pancreatic CSCs into NOD/SCID/IL2Rgamma mice, isolated from human pancreatic tumors via modulation of Sonic hedgehog-GLI pathway. Despite reduced expression of sHH components, sulforaphane also inhibited pluripotency maintaining transcription factors and markers of angiogenesis including vascular endothelial growth factor (VEGF) together with platelet-derived growth factor receptor alpha (PDGFRα). Interestingly, all of them are downstream targets for GLI transcription. Sulforaphane also reduced markers of EMT, in which Zinc finger E-box-binding homeobox 1 (Zeb-1) is included [83]. Moreover, sulforaphane inhibited CSCs properties and enhanced therapeutic efficacy of cisplatin in NSCLC through up-regulation of miR-214 which then targeted c-Myc in vitro and in xenografted nude mice [84]. Additionally, sulforaphane suppressed the growth of triple-negative breast cancer stem-like cells in vitro and in vivo via modulation of stem-related embryonic oncogene CRIPTO-1/TDGF1 (CR1) and its homologue CR3. Despite the crypto-modulated pathway, sulforaphane decreased expression of various stem cell markers [85]. PEITC is an effective inhibitor of colorectal CSCs by targeting Wnt/β-catenin pathway in vitro. PEITC reduced the size and number of cell spheroids and expression of CSCs markers and suppressed colony formation and proliferation indicating the repression of self-renewal ability [77]. PEITC also suppressed pluripotency factors, self-renewal capacity, and clonogenicity of CSCs in vitro CSCs model derived from colon cancer cells and in a mouse xenograft model injected with EpCAM-expressing cells [86].

Diallyl trisulfide (DATS) is a garlic derived organosulfur suggested to possess anticancer properties. DATS reduced tumorsphere formation, decreased CSCs markers expression, inhibited proliferation and induced apoptosis via inhibition of Wnt/β-catenin pathway and its target genes in colorectal cancer cell line [87]. Similarly, DATS inhibited the viability of CSCs, decreased expression of CSCs markers, inhibited proliferation and induced apoptosis in human breast cancer cell line also via inhibition of Wnt/ β-catenin pathway [88].

#### 3.1.2. Plant-Derived Functional Foods

Anti-cancer research demonstrated benefits of phytochemicals combinations over isolated phytochemicals [89]. Plant-derived functional foods contain various bioactive compounds, and therefore are effective agents targeting CSCs. Several studies evaluated anti-CSCs effectiveness of plant-functional foods in preclinical research. Green algae *Capsosiphon fluvescens* glycoprotein downregulated the Wnt-1 signaling pathway in human gastric cell line, and therefore inhibited gastric cancer cell migration [90]. Anticarcinogenic effects of plant derived functional foods were evaluated in the chemopreventive models of experimental rat mammary carcinogenesis. Administration of *Origanum vulgare* L. in diet in the lower dose (0.1%) suppressed expression of CD24 by 34% and by 57% in the higher dose (1%). Moreover, the level of expression of EpCAM was decreased by 14% and 10% respectively. Furthermore, dietary administration of *Syzygium aromaticum* L. in high dose (1%) showed decrease in expression of CD24 and CD44 and increase in expression of ALDH1. These effects on CSCs were associated with significant chemopreventive activity in both studies [91,92]. Pomegranate (*Punica granatum* L.) is a fruit rich in nutrients and bioactive phytochemicals [93]. A pomegranate emulsion was found to possess chemopreventive properties against DMBA-induced mammary tumorigenesis in rats via disruption of Estrogen Receptor and Wnt/β-catenin signaling pathways [94]. Further investigation revealed that preventive effects of pomegranate extract in DMBA-evoked mammary carcinogenesis involve anti-inflammatory regulation of two interrelated pathways NF-κB and Nrf2 [93], and this mechanism may be interrelated with CSCs signaling. *Trianthema portulacastrum* L. is an exotic plant exhibiting various pharmacological properties including antibacterial, antifungal, anti-inflammatory or antioxidant effects. *T. portulacastrum* extract (TPE) was found to prevent DMBA-induced breast carcinogenesis by anti-inflammatory mechanism mediated via modulation of NF-κB and Nrf signaling pathways [94]. Moreover, extract of *Geissospermum vellosii* also known as Pao Pereira, inhibited pancreatic CSCs via modulation of Wnt/β-catenin in vitro and in vivo [95]. Similarly, pancreatic CSCs were inhibited by extract of traditional African plant *Rauwolfia vomitoria* in vivo and in vitro also via modulation of Wnt/β-catenin signaling pathway [96]. Significantly, Chinese bayberry (*Myrica rubra*) leaf proanthocyanidins (BLPs) containing epigallocatechin-3-O-gallate (EGCG) as their terminal and major extension units exhibited inhibitory effects on chemotherapy-resistant OVCAR-3 spheroid cells via modulation of cell viability and sphere and colony formation. Furthermore, BLPs also inhibited self-renewal abilities of CSCs via targeting Wnt/β-catenin signaling pathway [97]. The anticancer benefits of three marine brown seaweed polyphenol extractions including *Hormophysa triquerta* (HT-EA), *Spatoglossum asperum* (SA-EA) or *Padina tetrastromatica* (PT-EA) were explored utilizing pancreatic cancer (PC) stem cells grown ex vivo and mouse model of residual-PC. Results of the study demonstrated the ability of these extracts to target signaling pathways playing critical role in the regulation of EMT, pluripotency and maintenance of CSCs after first-line therapy [98]. Water extract of *Gynura divaricata* (GDE) was found to target liver CSCs in a moderate to weak level and to sensitize Huh7 cell to cisplatin therapy by regulation of Wnt/β-catenin pathway and target genes [99].

Moreover, the efficacy of resveratrol (RSV) in combination with grape seed extract (GSE) was investigating in isolated human colon CSCs in vitro and in an azoxymethane-induced mouse model of colon carcinogenesis in vivo. RSV-GSE suppressed Wnt/β-catenin and induced mitochondrial-mediated apoptosis of CSCs [100]. A summary evaluating the anti-CSCs mechanisms of phytochemicals (isolated or mixtures) is shown in Table 3.

### 3.2. Clinical research

Anti-CSCs potential of dietary phytochemicals (isolated or mixtures) was investigated in several previously mentioned preclinical studies [43,49,52,66,67,69,70,71,72,74,75,77,78,79,80,83,84,85,86,87,88,90,91,92,93,94,95,96,97,98,99,100]. Significantly, anticancer properties of phytochemicals were evaluated in several clinical trials. Firstly, a phase I pilot study on patients with colon cancer was conducted to evaluate effects of a low dose of resveratrol formulation and resveratrol-containing freeze-dried grape powder (GP) on Wnt signaling in the colon. Results of the trial are based on the expression of Wnt target genes. Resveratrol/GP inhibited Wnt target gene expression in normal colon mucosa; however did not inhibit the Wnt pathway in colon cancer tissue [101]. Moreover, a phase 2 randomized, placebo-controlled trial was conducted on 59 subjects diagnosed with urothelial bladder cancer. The aim of the trial was to investigate whether daily dose of genistein in the form of purified soy extract G-2535 for 14 to 21 days before surgery alter molecular pathways in bladder epithelial tissue. The primary outcome of the study was inhibition of EGFR phosphorylation in a dose of 300mg/d. However, no significant changes were observed in expression of COX-2, Ki67, caspase-3, Akt or p-Akt [102]. Furthermore, a randomized clinical trial on 35 colorectal cancer patients, who were daily administrated 900 mg of ellagitannins-containing pomegranate extract (PE) showed the expression of CRC-genes in normal and cancerous colon tissue was evaluated before (biopsies) and after (surgical specimens) 5–35 days of supplementation. Tissues were also obtained from a control group of patients with no supplementation. The consumption of the PE was significantly associated with a counterbalance effect in the expression of CD44, β-catenin, p21 or others suggesting that the intake of PE modulated the impact on gene expression in a gene- and tissue-specific manner [103]. While searching in the clinical trial database [104], we found few clinical trials which may be partially related to CSCs. A prospective phase II study was initiated in 2015 with the aim to investigate the effect of Fursultiamine, a derivate of vitamin B, combined with concurrent chemo/radiotherapy in esophageal cancer patients (ClinicalTrials.gov Identifier: NCT02423811). Moreover, pancreatic ductal adenocarcinoma (PDA) stem cells were target of a pilot study initiated in 2013 with the aim to find whether the application of freeze-dried broccoli sprouts lead cancer inhibition in patients with advanced PDA (ClinicalTrials.gov Identifier: NCT01879878). However, no results of these clinical trials are available at this time.

**Figure 3:**
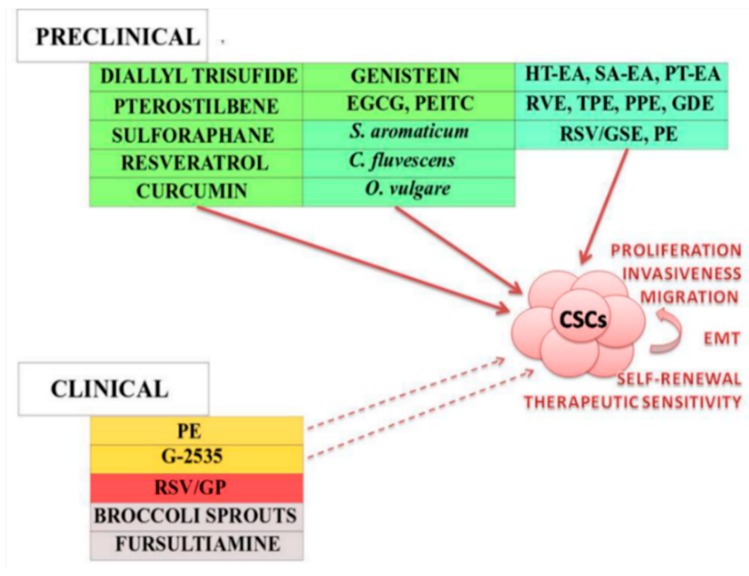
Dietary phytochemicals targeting CSCs in preclinical and clinical cancer research.

Figure 3 shows an overview of dietary phytochemicals (isolated or mixtures) in preclinical or clinical research and their effects on CSCs. Finally, there is number of clinical trials evaluating plant derived foods or dietary supplements in anticancer research; however, we were not successful in finding clinical trials focusing on CSCs specifically.

Isolated dietary phytochemicals including diallyl trisulfide, pterostilbene, sulforaphane, resveratrol, curcumin, genistein, epigallocatechin-3-gallate (EGCG), phenethyl isothiocyanate (PEITC) and plant functional foods including *S. aromaticum, C. fluvescens, O. vulgare,* Chinese bayberry leaf proanthocyanidins (BLPs) and extracts of pomegranate, *Trianthema portulacastrum, Gynura divaricata, Hormophysa triquerta* (HT-EA), *Spatoglossum asperum* (SA-EA), *Padina tetrastromatica* (PT-EA) and resveratrol in combination with grape seed extracts (GSE) demonstrated anticancer properties via targeting CSCs-mediated pathways and thus modulating CSCs proliferation, invasiveness, migration, self-renewal, EMT and sensitivity to therapeutic approaches in preclinical research. The data evaluating effects of dietary phytochemicals in clinical research were insufficient. Ellagitannins-containing pomegranate extract (PE) and purified soy extract (G-2535) may modulate CSCs signaling at least partially. Resveratrol formulation and resveratrol-containing freeze-dried grape powder RSV/GP did not exhibit any prosperous effects in inhibition of CSCs pathways in cancer tissue. Clinical trials evaluating anticancer effects of broccoli sprouts and fursultiamine were initiated in 2013 and 2015, however no results were reported for these studies.

## 4. Conclusion and Future Perspectives

There is great evidence suggesting that aberrant regulation of CSCs signaling pathways may lead to deregulation of self-renewal, apoptosis, proliferation, and importantly resistance to anti-cancer therapy. Considering the cancer research, phytochemicals (isolated or mixtures) are suggested to possess antioxidant, antiproliferative, and anticancer properties and also to have the ability to target aberrantly regulated signaling of CSCs. Importantly, the use of plant derived compounds is associated with no or very little adverse events. Phytochemicals are thought to modulate various signaling pathways of CSCs. Cross talk between these pathways influence self-renewal, differentiation, EMT, therapy resistance and other pro-cancer mechanism associated with stem-like cells. Here we summarized the current state of the anticancer effectiveness of different plant-derived dietary phytochemicals in preclinical and clinical research. In vitro and in vivo preclinical studies indicated significant anticancer effects of dietary phytochemicals mediated by CSCs targeting via modulation of signaling pathways, including Wnt, Notch, Hedgehog, or other, as well as via regulation of mechanisms involved in the processes of apoptosis or drug resistance. Based on the comparative preclinical oncology studies, functional foods (characterized by the presence of mixture of phytochemicals) are suggested to exhibit better anti-cancer activities (including the anti-CSCs properties) when compared to isolated phytochemicals. Importantly, each of the preclinical studies included in our review is specific in its aims and uses specifically designed methods. However, individual processes in the cell and therefore processes of carcinogenesis are complex and interconnected. Nevertheless, it would be beneficial to find out if there are associations or discrepancies between studies dealing with the same type of cancer, cell line, model, phytochemical or specific pathway responsible for anti-CSCs effects of particular substance. After all, more specific and comparative studies are needed for such analysis. Despite numerous preclinical studies, clinical research in this area is significantly lagging behind and only a few trials could be identified. On the contrary, we have encountered a large number of clinical studies focused on how are CSCs influenced by synthetic drugs; however, evidence of plant-derived foods or other dietary supplements as anti-CSCs agents is lacking. In conclusion, we emphasize the significant anti-cancer effects of dietary phytochemicals on CSCs in a wide range of cancer types via influencing multiple signaling mechanisms, and thus demonstrating the urgent need for their in-depth investigation in clinical research.

## Figures and Tables

**Table 1:** Markers of CSCs in various tissues.

Cancer Type	Marker	References
Brain cancer	CD34^+^/CD38^−^/CD133^+^/CD44^+^	[6,23]
Breast cancer	CD44^+^/CD24^−^/Lineage^-^/ALDH1^+^/ EpCAM^+^	[18,20,23]
Colon cancer	CD133^+^/CD44^+^/CD166^+^/ ALDH1^+^/LGR5^+^/EpCAM^+^	[6,18,19]
Leukemia	CD34^+^/CD38^−^/CD90^−^	[6,18,19]
Liver	CD133^+^/CD90^+^/EpCAM^+^	[6]
Lung	CD133^+^/CD44^+^/CD90^+^	[2,18]
Ovary	CD44^+^/ALDH1^+^/CD133^+^	[6,18]
Pancreas	CD44^+^/CD24^+^/CD133^+^/EpCAM^+^	[18,23]

Explanatory notes: + presence; ^−^ absence. Abbreviations used: ALDH1-Aldehyde dehydrogenase 1; CD24/34/38/44/90/133/166-Cluster of Differentiation 24/34/38/44/90/133/166; EpCAM-Epithelial Cell Adhesion Molecule.

**Table 2:** Cancer stem cells signaling pathways aberrantly regulated in selected malignancies.

Signaling Pathway	Cancer Type	Mechanism of Action	References
Notch	Brain cancer	↑Notch1 ↑JAG1 ↑DLL1	[6,40,44]
	T cell acute lymphoblastic leukemia	↑ Notch1	
	Breast cancer	↑Notch1 ↑JAG1,	
	Pancreatic cancer	↑Notch1 ↑Notch3 ↑Jag1 ↑Jag2 ↑Hes1	
	Non-Small Lung Cancer	↑ Notch3	
Wnt/β-catenin	Breast cancer	↑LEF-1 ↑TCF-4 ↑cyclin D1 ↑β-catenin ↓SFRP	[6,40,41,47]
	Colorectal carcinoma Brain tumor Prostate cancer	Mutations in APC/β-catenin site	
	Hematologic cancer Skin cancer Lung cancer	↓ WIF-1 ↓SFRP-1 ↓ DKK ↓AXIN2	
Hedgehog	Colon cancer	↑sHH ↑GLI2	[6,40]
	Medulloblastoma predisposition	Mutations in PTCH1	
	Myeloma	↑SMO ↑GLI1	
	Glioma	↑GLI1 ↑SHH ↑PTCH1	
PI3K/Akt/mTOR	Gastric cancer	↑Akt1	[19]
	Ovarian cancer Pancreatic cancer	↑Akt2	
	T cell acute lymphoblastic leukemia Melanoma Endometrial carcinoma Prostate cancer Glioblastoma	Mutations in PTEN	
JAK/STAT	Breast cancer Gastric cancer Glioblastoma	↑STAT3	[53,54,40]

Explanatory notes: ↓decrease; ↑increase.

**Table 3:** Anticancer mechanisms of dietary phytochemicals (isolated or mixtures) targeting CSCs.

Phytochemical (Isolated or Mixture)	Cell Line/Animal Model	Mechanism	References
EGCG	A549, H1299	↓β-catenin ↓CD133 ↓CD44 ↓ALDH1A1 ↓Nanog ↓Oct4	[66]
EGCG/ EGCG and cisplatin	HNSC CSCs BALB/c nude mice	↓Oct4 ↓Sox2 ↓Nanog ↓CD44 ↓ABCC2 ↓ABCG2	[67]
Resveratrol	MCF-7, SUM159 NOD/SCID xenografted mice	→autophagy ↓Wnt/β-catenin	[69]
	GBM2, GBM7, G144, G179, G166, GliNS2, GBM04	↓β-catenin ↓c-Myc ↓Twist1 ↓Snail1	[70]
	MNNG/HOS. MG-63, hFOB1.19	↓JAK2/STAT3 ↓CD133	[71]
Pterostilbene	MCF7, MDA-MB-231	↓NF-κB ↓Twist1 ↓vimentin ↑E-cadherin	[72]
	HCC Mahlavu	↓c-Myc ↓COX-2 ↓vimentin ↓CXCR4 ↓Twist1	[43]
Genistein	MCF-7 Nude mice	↓SMO ↓GLI1	[74]
	SKOV3 Nude mice	↓CD 163 ↓p-STAT3 ↓IL-10 ↑IL-12 ↓CD133 ↓CD44	[52]
	GCSLCs	↓ Twist1 ↓N-cadherin ↑E-cadherin ↓CD133 ↓CD44 ↓ALDH1	[75]
Curcumin	BL41-3, Ramos, DG-75, THP-1	↓ALDH+ cells ↓GLI1 ↓Notch1 ↓cyclin D1	[78]
	MCF-7, MDA-MB-231 Athymic mice	↓ABCG2 ↓ABCC1	[79]
	U87, T98G U87-implanted nude mice	↓sHH ↓SMO ↓GLI1 ↓cyclin D1 ↓Bcl-2 ↓FoxM1 ↑Bax/Bcl-2 ratio	[49]
	DU-145	↓cyclin D1 ↓CDK2 ↓Bcl-2 ↑p21 ↑p27 ↑p53	[80]
Sulforaphane	NOD/SCID/IL2Rgamma mice	↓SMO ↓GLI1 ↓GLI2 ↓Nanog ↓Oct-4 ↑Bcl-2 ↓Zeb-1 ↓E-cadherin ↓VEGF ↓PDGFRα	[83]
	BalbC/nude mice	↓CR1 ↓CR3 ↓Nanog ↓ALDHH1A1 ↓Wnt3 ↓Notch4	[85]
	BEAS-2B, H460, H1299, A549	↓c-Myc	[84]
Phenethyl isothiocyanate	DLD-1 SW480	↓size/number of cell spheroids ↓CD133+	[77]
	293T, NCCIT, HCT116 Xenograft model	↓Oct4 ↓Sox-2 ↓Nanog	[86]
Diallyl trisulfide	SW48, DLD-1	↓β-catenin ↓c-Myc ↓cyclin D1	[87]
	MCF-7, SUM159	↓CD44 ↓ALDH1A1 ↓Nanog ↓Oct4	[88]
*Capsosiphon fulvescens*	AGS	↓Wnt-1 ↓β-catenin → G0/G1 arrest	[90]
*Origanum vulgare*	NMU-induced rat mammary carcinogenesis	↓CD24 ↓EpCAM	[91]
*Syzygium aromaticum*	NMU-induced rat mammary carcinogenesis	↓CD24 ↓CD44 ↑ALDH1	[92]
Pomegranate extract	DMBA-induced rat mammary carcinogenesis	↓ER-α:ER-β↓β-catenin ↓cyclin D1 ↓COX-2 ↑Nrf2 IκBα degradation and NF-κB translocation blockage	[93]
*Trianthema portulacastrum* extract	DMBA-induced rat mammary carcinogenesis	↓COX-2 ↑Nrf2 IκBα degradation and NF-κB translocation blockage	[94]
Pao Pereira extract	PANC-1, MIA PaCa-2, AsPC-1, HPAF-II, BxPC-3 in immunocompromised mice	↓Nanog ↓β-catenin	[95]
Rauwolfia vomitoria extract	PANC-1, AsPC-1, HPAF-II, BxPC-3 and MiA PaCa-2 in immunocompromised mice	↓Nanog ↓β-catenin	[96]
Chinese bayberry leaf proanthocyanidins (BLPs)	OVCAR-3	↓β-catenin ↓cyclin D1 ↓c-Myc →G1 arrest	[97]
HT-EA, SA-EA, PT-EA	Panc-1, MiaPaCa-2, Panc-3.27, and BxPC-3 Athymic NCr-nu/nu nude mice	↓Nanog ↓Oct-4 ↓Sox2 ↓N-cadherin	[98]
Water extract of *Gynura divaricata* (GDE)	Huh7, Hep3B	↓β-catenin	[99]
Resveratrol (RSV) and grape seed extracts (GSE)	Human colon CSCs Azoxymethane-induced mice	↓ nuclear translocation of β-catenin ↓c-Myc ↓cyclin D1 ↑p53 ↑Bax/Bcl-2	[100]

Explanatory notes: ↓ decrease; ↑ increase; → induction. Abbreviations used: ABCC1-ATP Binding Cassette Subfamily C Member 1; ABCC2-ATP Binding Cassette Subfamily C Member 2; ABCG2-ATP Binding Cassette Subfamily G Member 2; ALDH1A1-Aldehyde Dehydrogenase 1 Family Member A1; Bcl-2-B-cell Lymphoma Protein Family CD136/133/44/24, Cluster of Differentiation 136/133/44/24; CDK2-Cyclin-dependent Kinase 2; c-Myc, MYC protoonkogene; COX-2-Cyclo- oxygenase 2; CR1-CRIPTO-1/TDGF1, Teratocarcinoma-derived Growth Factor 1, CR3, CRIPTO-3/TDGF1P3, Putative Teratocarcinoma-derived Growth Factor 3; CXCR4-Chemokine Receptor Type 4; EpCAM-Epithelial Cell Adhesion Molecule; ERα-Estrogen Receptor Alfa; ERβ-Estrogen Receptor Beta; FoxM1-Forkhead Box Protein M1; GLI1/2-Zinc Finger Protein 1/2; IL-10/12-Interleukin 10/12; JAK-Janus kinase; NF-κB-Nuclear Factor Kappa-light-chain-enhancer of Activated B cells; Notch1-4-Notch receptors; NRF2-Nuclear Factor Erythroid 2–related Factor 2; p21-Cyclin-dependent Kinase Inhibitor 1; p27-Cyclin-dependent Kinase Inhibitor 1B; p53-Tumour Protein p53; PDGFRα-Platelet-derived Growth Factor Receptor Alpha; pSTAT3-Phospho-Signal Transducer and Activator of Transcription 3; sHH- Sonic Hedgehog; SMO- Transmembrane protein SMOOTHENED; Snail1-Zinc Finger Protein; STAT-Signal Transducer and Activator of Transcription; Twist1-Twist family BHLH Transcription Factor 1/gene; VEGF-Vascular Endothelial Growth Factor; Zeb-1-Zinc finger E-box-binding Homeobox 1.
